# Evaluation of two stereophotogrametry software for 3D reconstruction of virtual facial models

**DOI:** 10.1590/2177-6709.27.3.e2220230.oar

**Published:** 2022-07-04

**Authors:** Lorena Basilio CHAVES, Taliane Lima BARBOSA, Caroline Pelagio Maués CASAGRANDE, David Silveira ALENCAR, Jonas CAPELLI, Felipe de Assis Ribeiro CARVALHO

**Affiliations:** 1 Universidade do Estado do Rio de Janeiro, Departamento de Ortodontia (Rio de Janeiro/RJ, Brazil).; 2 Universidade Federal Fluminense, Departamento de Ortodontia (Niterói/RJ, Brazil).

**Keywords:** Diagnostic imaging, Photogrammetry, Anatomy

## Abstract

**Objective:**

The present study aimed to evaluate the accuracy of 3D facial soft tissue virtual models produced by two photogrammetry softwares (AgiSoft Photoscan and 3DF Zephyr Free), when compared to those created by cone beam computed tomography (CBCT).

**Methods:**

Ten patients were submitted to two sequences of photographs performed with a DSLR camera (with and without the aid of a ring flash) and CBCT scans. Each photo series for each patient was processed with the softwares, and at the end, five models of each patient were generated: 1) CBCT, 2) AAL (Agisoft Ambient Light), 3) AFL (Agisoft Flash Light), 4) ZAL (Zephyr Ambient Light), and 5) ZFL (Zephyr Flash Light). Color coded maps and root-mean-square (RMS) distances were used to compare the photogrammetry models to the CBCT ones.

**Results:**

One sample *t-*test showed significant differences between all methods *versus* CBCT. The worst results were seen in the ZAL group (discrepancies up to 5.17mm), while the best results were produced by AAL group (discrepancies up to 2.11mm).

**Conclusions:**

It can be concluded that this type of virtual facial models are reasonably accurate, although not perfect, and considering its lower biological and financial cost, they may play an important role in specific situations.

## RESUMO

**Objetivo:**

O presente estudo teve como objetivo avaliar a acurácia de modelos virtuais 3D de tecidos moles faciais produzidos por dois *softwares* de fotogrametria (AgiSoft Photoscan e 3DF Zephyr Free), em comparação aos gerados por tomografia computadorizada de feixe cônico (TCFC).

**Métodos:**

Dez pacientes foram submetidos a duas sequências de fotografias realizadas com câmera DSLR (com e sem auxílio de *ring flash*) e a TCFC. Cada série de fotografias de cada paciente foi processada com os *softwares* e, ao fim, foram gerados cinco modelos de cada paciente: 1) TCFC; 2) AAL (Agisoft Ambient Lighting); 3) AFL (Agisoft Flash Light); 4) ZAL (Zephyr Ambient Lighting); e 5) ZFL (Zephyr Flash Light). Mapas codificados por cores e distâncias quadráticas médias (RMS) foram usados para comparar os modelos de fotogrametria com os de TCFC.

**Resultados:**

O teste *t* para uma amostra mostrou diferenças significativas entre todos os métodos *versus* a TCFC. Os piores resultados foram observados no grupo ZAL (discrepâncias de até 5,17 mm), enquanto os melhores resultados foram produzidos pelo grupo AAL (discrepâncias de até 2,11 mm).

**Conclusões:**

Pode-se concluir que esse tipo de modelos faciais virtuais é razoavelmente preciso, embora não sejam perfeitos; e, considerando-se seu menor custo biológico e financeiro, podem desempenhar um papel importante em situações específicas.

## INTRODUCTION

Adequate planning is key for orthodontic success, prognosis definition, as well as for treatment duration prediction. Comprehensive planning demands diagnostic techniques to identify aesthetical, functional and anatomical aspects. However, a divergence in the diagnosis of orthodontic problems among professionals is still very frequent.^1^ Facial analysis is a valuable tool for quantitative and qualitative assessment, and its application is increasingly demanded by professionals and patients.^2^ ^,^ ^3^

At the beginning of the 20th century, Angle stated that the orthodontist would be able to classify malocclusion only by facial evaluation.^4^ But it was only in 1999 that the importance of soft tissue assessment in diagnosis and treatment planning was highlighted, and its use was advocated, recognizing the face as a determining factor in orthodontic planning. Currently, facial aesthetics is being increasingly considered in the decision making process of teeth extractions, sagittal and asymmetry camouflage, as well as orthognathic surgery.^6^ ^-^ ^10^

Most popular facial analysis methods include photographs and cephalometric measurements based on two-dimensional (2D) images. These have inherent limitations, such as a significant amount of radiographic projection error, distortion, inaccurate duplication of measurements, significant variation in the position of reference points, among others.^11^ ^,^ ^12^

After the introduction of three-dimensional (3D) technologies in dentistry, 3D imaging systems are increasingly being used instead of 2D ones, especially in cases of craniofacial deformities. In fact, three-dimensional image provides more detailed and realistic information on craniofacial soft and hard tissues, and allows for easier, faster and more reliable analysis, even though some methods still have limitations.^13^ ^-^ ^15^

Although Cone-Beam Computed Tomography (CBCT) could be considered the most accurate 3D imaging method for diagnosis and follow-up of orthodontic treatment results, its use is limited due to its high costs and, mainly, due to the exposure of patients to ionizing radiation.^16^ ^,^ ^17^ Laser scanning, stereophotogrammetry (SPG), video-image, structured light scanners among other methods for obtaining 3D images without the use of radiation have already been proposed.^18^ ^-^ ^21^

SPG scanners like the 3dMDface system and the Di3D system are able to generate very realistic and accurate 3D facial models.^22^ ^-^ ^24^ However, these scanners need a dedicated and costly hardware, which may reduce their clinical application. Therefore, the search for alternative 3D facial models acquisition methods, with lower financial and biological costs is relevant in the current orthodontic scenario. In this context, some methods are promising for the clinical popularization of facial scanning, such as the Microsoft Kinect scanner, which generated average results, with a low cost hardware.^25^

A viable alternative might be software that allows the 3D reconstruction from a series of 2D pictures, without the need of any specialized hardware rather than a photo camera, such as Agisoft Photo Scan^©^ (Agisoft - St. Petersburg, Russia) or Zephyr 3D^©^ (3Dflow SRL - Verona, Italy). 

These softwares could allow the spread of 3D facial acquisition technology, as cameras are easy assets in today’s clinics use and the software’s licenses are considerably cheaper than the costs involved in owning and maintaining a 3D facial scanner or CBCT device, but little is presented in the literature regarding their precision.

This study aims to evaluate the accuracy of two SPG softwares (Agisoft Photo Scan, AgiSoft 2018, St. Petersburg, Russia and 3DF Zephyr, 3DFLOW SRL Udine, Italy) in obtaining three-dimensional facial models, compared to traditional CBCT soft tissue models.

## MATERIAL AND METHODS

Sample size calculation was performed using the GPower3.1 software (University of Kiel, Germany) with a one sample *t*-test. Based on parameters of a study with similar methodology^25^ and considering a power of hypothesis test of 80%, a level significance of 0.05, to detect a difference in measurements of 2mm with a standard deviation of 2.03, at least eight volunteers would be needed for this research.

Ten patients were selected for this study, five males and five females, with a mean age of 24.4 years, who needed a full-head CBCT scanning at the beginning of orthodontic treatment. Exclusion criteria involved patients who were already using orthodontics appliances, with syndromes and /or craniofacial deformities, with a beard and/or mustache, with tattoos on the face, or with severe facial asymmetries. The selected patients agreed to participate with the use of their images in the study and signed an informed consent form. This study was approved by the Research Ethics Committee of the University, under the number 3.177.721.

CBCT were obtained with the Classic iCAT (Image Sciences, Hatfield, PA, USA) scanner, with the following parameters: 0.3-mm isometric voxel, 40 seconds of exposure, extended field of view (22 cm high), with the patient sitting and in centric occlusion (CO). All the participants were asked not to wear makeup, beard or mustache, earring, glasses, facial products or any type of accessories. They were also instructed to keep the head still and positioned in a way to have the Camper plane parallel to the ground.

Immediately after the CBCT acquisition, each patient underwent two photo sessions, performed by the same researcher in an indoor environment with clear walls and adequate lighting, following the same instructions given during the CBCT scanning.

**Figure 1:**
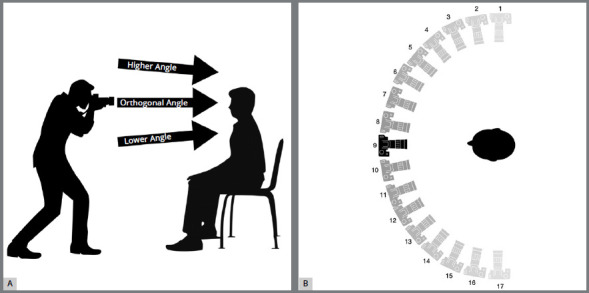
Scheme illustrating the photo acquisition process used in the research: A) Side view, showing the three photography angles taken at each point; B) Top view showing the 17 points around the patient, where the three photography angles were executed.

Each series of photographs consisted of 51 photos taken at 17 pre-established reference points, with similar distances between them, marked on the floor (180º around the patient’s face, who was positioned in the center of the semicircle formed with the markings) as shown in Figure 1. At each point, the researcher took three photos at three different angles, one with a higher view of the patient’s head, the second with an orthogonal view and the third with a lower view. This sequence was implemented taking into account the particularities of the softwares described in their respective manuals.

The first photo session was only performed with ambient light (Canon EOS REBEL T3i with Canon Compact - Macro Lens EF 50mm 1:2.5). The exposure settings were set as following: 8.0MP resolution (3456 x 2304), t = 1/60, f = 5.6 and ISO 1600, and then another session was performed with the same positioning and camera model, but using an auxiliary lighting (Canon MACRO RING LITE MR -14EX II set to ETTL mode, E.V. = +0.7, RATIO A:B = 1:1) attached to the camera lens. In this session, the exposure settings were set as following: 8.0MP resolution (3456 x 2304), t = 1/80, f = 8.0 and ISO 800.

**Figure 2:**
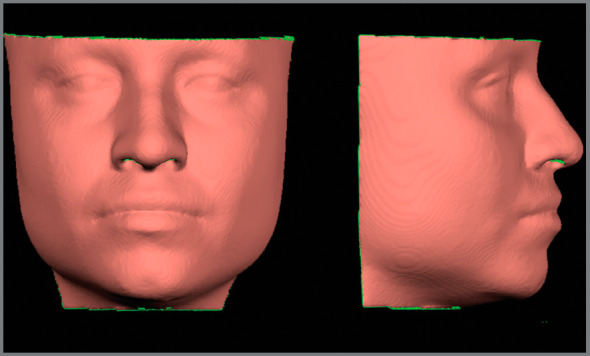
Limit of facial soft tissue models obtained from CBCT, exemplifying the evaluated region.

All CBCT scans were imported to Dolphin Imaging 11.95 software in order to build a soft tissue facial 3D surface model that could be exported in the STL format. These models were limited, excluding regions of the face that would not be evaluated and included only the region delimited, in length, by porium on each side of the face; and in height, from the superior line of the orbits to the lower limit of the mandible, as shown in Figure 2.

Two distinct SPG softwares were used to allow the conversion of each patient´s photo series in a soft tissue facial 3D surface model that could also be exported in the STL format (Agisoft PhotoScan Professional Edition v.1.2.4 and 3DF Zephyr Free Edition v.4.352). The limits considered for the photogrammetry models were the same used for the tomographic models generation.

**Figure 3:**
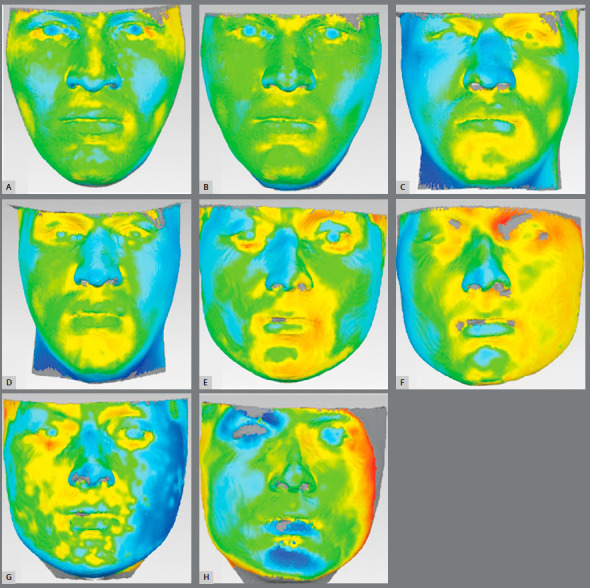
Color map models with the best and worst results within the sample: **A**) best AFL x CBCT; **B**) best AAL x CBCT; **C**) best ZFL x CBCT; **D**) best ZAL x CBCT; **E**) worst AFL x CBCT; **F**) worst AAL x CBCT; **G**) worst ZFL x CBCT;**H**) worst ZAL x CBCT.

Five STL files of each patient’s face (1 - CBCT group, created by the CBCT; 2 - Agisoft Ambient Lighting group - AAL, created by the Agisoft PhotoScan using the ambient light photo series; 3 - Agisoft Flash Light group - AFL, created by the Agisoft PhotoScan using the ring flash light photo series: 4 - Zephyr Ambient Light group - ZAL, created by the 3DF Zephyr using the ambient light photo series: and 5 - Zephyr Flash Light group - ZFL, created by the 3DF Zephyr using the ring flash light photo series) were imported into the Geomagic Qualify 2013 (ResearchTriangle Park, NC) and a best fit superimposition was done for each group, always considering the CBCT facial models as a reference, generating at the end a total of four comparisons (AAL *vs* CBCT; AFL *vs* CBCT; ZAL *vs* CBCT; and ZFL *vs* CBCT) with color map models, exemplified in Figure 3.

**Figure 4:**
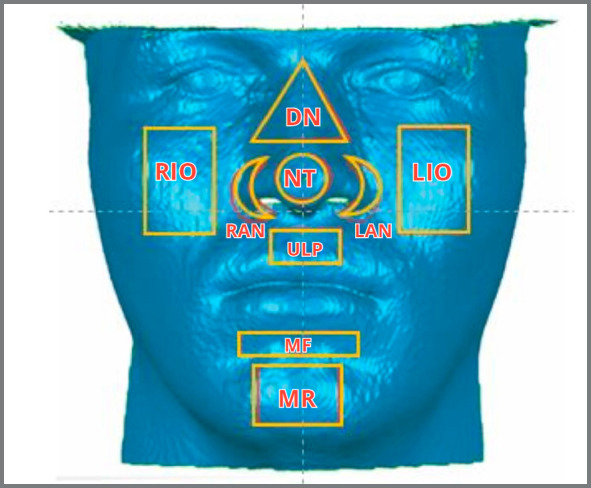
Example of the anatomical regions of interest (ARIs) used to evaluate the tested models.

The face was then divided into nine anatomical regions of interest (ARIs): 1) tip of the nose (NT); 2) right ala-nostril sill (RAN); 3) left ala-nostril sill (LAN); 4) dorsal nose (DN); 5) upper lip philtrum (ULP); 6) mentolabial fold (MF); 7) mental region (MR); 8) right infraorbital region (RIO); and 9) left infraorbital region (LIO), as seen in Figure 4.

After the ARI cropping, files for each of them were exported to STL format and then converted to IV format. MeshValmet^®^ 3.0 software (https://www.nitrc.org/projects/MeshValmet), allowed the quantification of the differences between the ARI for the four previously explained comparisons using the RMS values.^26^

## STATISTICAL ANALYSIS

Normal distribution was verified by means of Shapiro-Wilk test. Descriptive statistics of the recorded RMS values of each comparison for each ARI is shown in Table 1. One sample *t*-test was used to verify if the recorded differences were statistically different from 0. The level of significance was set at 0.05.

**Table 1:** Descriptive analysis of the results obtained by groups.

Mean					Std. Deviation				95% Confidence Interval for Mean								***p*-value**			
									Lower bound				Upper bound							
	AFL vs CBCT	AAL vs CBCT	ZFL vs CBCT	ZAL vs CBCT	AFL vs CBCT	AAL vs CBCT	ZFL vs CBCT	ZAL vs CBCT	AFL vs CBCT	AAL vs CBCT	ZFL vs CBCT	ZAL vs CBCT	AFL vs CBCT	AAL vs CBCT	ZFL vs CBCT	ZAL vs CBCT	AFL vs CBCT	AAL vs CBCT	ZFL vs CBCT	ZAL vs CBCT
LAN	1.5	1.59	1.57	3.48	0.85	1.19	0.3	1.21	0.89	0.73	1.35	2.41	2.12	2.45	1.79	4.54	0	0.002	0	0
RAN	1.2	1.42	1.45	3.1	0.34	0.69	0.32	1.48	0.96	0.92	1.22	2.22	1.45	1.91	1.69	3.97	0	0	0	0
NT	2.2	2.11	3.17	5.17	1.49	1.36	1.07	1.27	1.13	1.13	2.39	4.25	3.27	3.08	3.94	6.08	0.001	0.001	0	0
DN	1.57	0.96	2.27	4.85	1.05	1.11	1.04	2.11	0.81	0.16	1.53	3.34	2.32	1.76	3.02	6.37	0.001	0.023	0	0
RIO	0.79	0.63	0.94	2.84	0.26	0.44	0.5	0.83	0.6	0.31	0.58	2.24	0.99	0.94	1.31	3.44	0	0.001	0	0
LIO	0.84	0.71	1.71	2.65	0.38	0.49	1.52	1.22	0.56	0.35	0.62	1.77	1.12	1.06	2.81	3.53	0	0.001	0.006	0
ULP	1.34	1.09	0.86	2.65	0.75	0.74	0.29	0.75	0.8	0.56	0.65	2.11	1.89	1.62	1.07	3.19	0	0.001	0	0
MR	1.1	0.9	1.43	2.92	0.67	0.69	0.89	1.67	0.62	0.4	0.79	1.72	1.58	1.4	2.07	4.12	0.001	0.003	0.001	0
MF	1.14	0.83	1.23	3.2	0.65	0.5	0.49	0.97	0.67	0.47	0.87	2.5	1.62	1.19	1.58	3.9	0	0.001	0	0

## RESULTS

The one sample *t*-test results showed that there was a statistically significant difference between the ARIs (all with *p*< 0.05) in all groups compared. Models generated in 3DF Zephyr showed poor accuracy. When comparing CBCT *vs* ZAL, the mean differences were <4mm for all ARI, except for DN and NT. Better results were found when comparing CBCT *vs* ZFL, where the mean differences were < 3.2mm.

**Figure 5:**
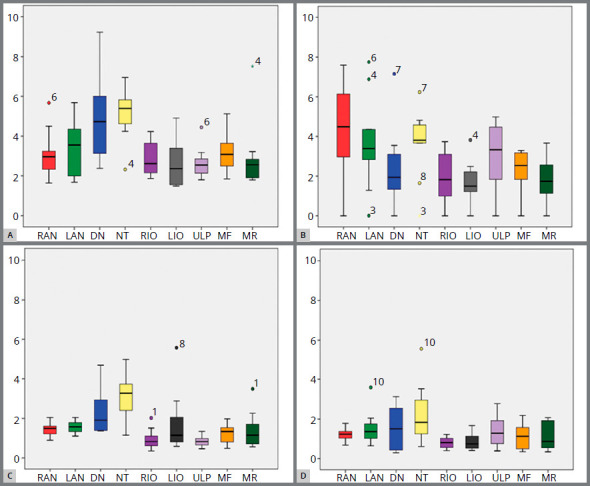
Boxplots illustrating results dispersion for: **A**) CBCT x ZAL; **B**) CBCT x AAL; **C**) CBCT X ZFL; **D**) CBCT X AFL.

Models generated in Agisoft showed acceptable accuracy for both groups (AAL and AFL). When comparing CBCT *vs* AAL, the mean differences were < 2.3mm. Comparing CBCT *vs* AFL, the mean difference was < 2.2mm (Fig 5 and Table 1).

## DISCUSSION

The evaluation of the models from AAL group showed differences in relation to the gold standard, with perceptible clinical relevance. Considering a difference of 2mm between AAL and CBCT methods as acceptable, only the tip of the nose showed a significant disagreement, which reinforces this method as promising. The nose was also the only critical region when evaluating AFL, with just NT and DN regions presenting discrepancies greater than 2 mm when compared to the gold standard, allowing subjective analysis by patients and orthodontists.

The 3DF Zephyr software presented poor results for the ZAL group and smaller disparities, when comparing the ZFL group to the CBCT. Maximum differences of up to 3mm were found when comparing ZAL *vs* CBCT for the following regions: RIO, LIO, ULP and MF. On the other hand, ZFL group showed 7 ARIs with differences smaller than 2mm, except for NT and DN; showing a similar performance to the AFL group. 

The nose region was the most critical for all the evaluated situations; being challenging to accurately represent its morphology with all the evaluated methods. This limitation is remarkable but doesn’t preclude its application in orthodontics since, the nose do not undergo major modifications following orthodontic treatment. This way, these softwares have shown promise for facial soft tissues evaluation when a tomographic scan is not justifiable due to its biological and/or financial cost.^16^

The worst accuracy was observed for all methods in the NT region. This fact may be explained due to its simpler anatomy, being a small and usually smooth region without any marked anatomical feature, which implies a more challenging scenario for the software’s algorithm to distinguish it from the background. 

This NT region is also hard to be reconstructed from CBCT scans, due to its delicate soft-tissue-only composition, which barely attenuates the X-ray and therefore compromises its acquisition. Because of this CBCT limitation (used in this paper as the gold standard), it is impossible to reliably quantify the photogrammetry inaccuracy in the NT region, and all the measured discrepancies probably represent a sum of the errors of the CBCT and the SPG. These results reinforce the findings of Maués et al,^25^ who observed inconsistent NT reconstructions from either Di3D and Microsoft Kinect generated 3D models. 

RIO and LIO presented differences smaller than 1mm in the AFL and AAL groups, which can be explained by the fact that they are broader regions and less prone to unwanted shadows. Such regions are often modified by specific orthodontic therapies such as maxillary protraction. Thus, monitoring these modifications with the SPG would be promising, since it could require a greater number of exams, contraindicating the use of CBCT, due to its biological and financial cost.

The regions of vermilion of the upper and lower lip were not measured in the present study, although they are areas of possibly greater alteration during orthodontic treatment. These regions were not evaluated due to the high variability of lip posture in the resting position among the two different timepoints (CBCT and photo sequence). Additionally, the inter-labial region is highly prone to unwanted shadows, a fact that could generate non-existent discrepancies during the three-dimensional reconstruction and later superimposition quantification.

In the present study, the photographs were taken indoors, with ambient light and, for a certain series of photos, with the aid of a ring flash. This fact may be limiting and determinant for the occurrence of shadows in certain ARIs, but it represents the ambient lightening of a typical dental office. New studies should be carried out in ambients with better lighting, to evaluate its influence on the quality of the generated models.

Another variable that could influence the models generated by the photo series is the quality of the camera sensor and the settings used. Since the methods evaluated by the present study are suitable for clinical follow up evaluation, the authors choice of a basic DSLR with ringflash was based on the hardware setup normally used by clinical dentists. Professional cameras, with their state-of-the-art sensors, could be able to compensate for the limited availability of light in the ambient, which would reduce artifacts generation in the two-dimensional images, making it easier, for the software, to identify landmarks to reconstruct the models. However, their higher prices would limit the diffusion of the proposed technique.

Likewise, especially in the 3DF Zephyr instruction manual, it is recommended to overlap 70-80% of surface in each photo, limiting the angles formed between them. It is advisable to make as many photographs as possible so that better final results may be obtained. However, this photo additions would increase the duration of acquisition, which could make the technique less viable. 

Since the purpose of this study wass to verify the accuracy of the methodology, the number of photos taken was chosen to use the maximum potential of the software. Due to the lack of methodologies reported in the literature to date, and the constant evolution of software programming, less photos could be tested in future papers. However, the method is applicable for clinical use, with 5-minute average execution time for each photo sequence.

Other scientifically proven methods that use SPG have the disadvantage of their high acquisition cost²³, and new facial reconstruction methods are being developed, turning SPG more practical,^25^ mainly by using mobile applications for images acquisition. However, these methods also need to be tested for accuracy, as there are no studies to this effect.

**Figure 6:**
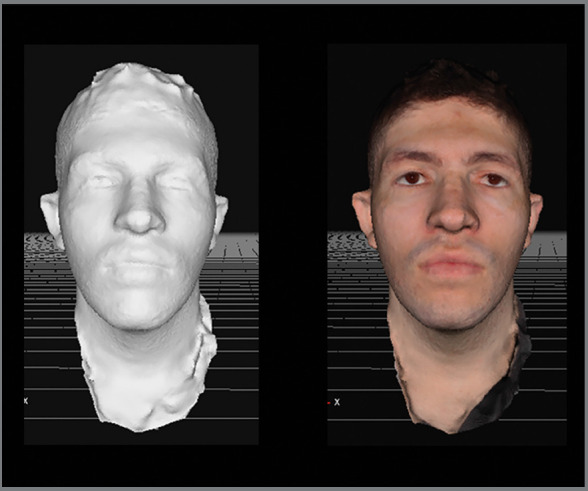
3D models of the same patient in three-dimensional (left) and textured (right) mesh.

The models obtained through software were generated in three-dimensional meshes that were compared to those produced by CBCT. However, the same models with texturing of facial tissues (Fig 6 ) are more pleasing to laypeople, and would allow subjective analyzes of patients submitted to the method,^3^ which could be a good tool for qualitative comparison of results and treatment follow-up.

## CONCLUSIONS

It can be concluded that the use of the 3D reconstruction method of facial soft tissues from photogrammetry in dentistry did not match the gold standard established by the CBCT, however, it is technically feasible to use the method proposed for periodic subjective evaluations during treatment, since its limitations are known. However, software evolution and further studies are needed to improve the method and enhance the quality of the models obtained.
